# Neoadjuvant Therapy of DOF Regimen Plus Bevacizumab Can Increase Surgical Resection Ratein Locally Advanced Gastric Cancer: A Randomized, Controlled Study

**DOI:** 10.1097/MD.0000000000001489

**Published:** 2015-10-23

**Authors:** Junxun Ma, Sheng Yao, Xiao-Song Li, Huan-Rong Kang, Fang-Fang Yao, Nan Du

**Affiliations:** From the Department of Medical Oncology, First Affiliated Hospital, Chinese PLA General Hospital, Beijing, China.

## Abstract

Locally advanced gastric cancer (LAGC) is best treated with surgical resection. Bevacizumab in combination with chemotherapy has shown promising results in treating advanced gastric cancer. This study aimed to investigate the efficacy of neoadjuvant chemotherapy using the docetaxel/oxaliplatin/5-FU (DOF) regimen and bevacizumab in LAGC patients.

Eighty LAGC patients were randomized to receive DOF alone (n = 40) or DOF plus bevacizumab (n = 40) as neoadjuvant therapy before surgery. The lesions were evaluated at baseline and during treatment. Circulating tumor cells (CTCs) were counted using the FISH test. Patients were followed up for 3 years to analyze the disease-free survival (DFS) and overall survival (OS).

The total response rate was significantly higher in the DOF plus bevacizumab group than the DOF group (65% vs 42.5%, *P* = 0.0436). The addition of bevacizumab significantly increased the surgical resection rate and the R0 resection rate (*P* < 0.05). The DOF plus bevacizumab group showed significantly greater reduction in CTC counts after neoadjuvant therapy in comparison with the DOF group (*P* = 0.0335). Although the DOF plus bevacizumab group had significantly improved DFS than the DOF group (15.2 months vs 12.3 months, *P* = 0.013), the 2 groups did not differ significantly in OS (17.6 ± 1.8 months vs 16.4 ± 1.9 months, *P* = 0.776. Cox proportional model analysis showed that number of metastatic lymph nodes, CTC reduction, R0 resection, and neoadjuvant therapy are independent prognostic factors for patients with LAGC.

Neoadjuvant of DOF regimen plus bevacizumab can improve the R0 resection rate and DFS in LAGC. These beneficial effects might be associated with the reduction in CTC counts.

## INTRODUCTION

Gastric cancer is one of the most common malignancy in Asian patients.^1^ Surgical resection is the curative treatment for early stage gastric cancer. However, > 60% of Chinese presented with locally advanced gastric cancer (LAGC) and had very poor prognosis after surgery.^2^ Adjuvant therapy has previously been used in gastric cancer but failed to improve survival in these patients.^3–5^ In recent years, several clinical trials have shown that adjuvant therapy can benefit patient survival after D2 surgery for gastric cancer. These trials used combinations of new drugs or different delivery time, which was different from their previous studies. The MAGIC study found that the 5-year survival rate was 38% in patients receiving perioperative adjuvant chemotherapy in comparison with 23% in those receiving surgery alone.^6^ Other studies have shown an increase in the 5-year survival rate up to 15% in patients treated with neoadjuvant chemotherapy.^7,8^ Neoadjuvant chemotherapy is increasing being used in patients with advanced gastric cancer.

Bevacizumab is a recombinant, humanized murine monoclonal antibody against vascular endothelial growth factor A (VEGF-A).^9^ Bevacizumab has been shown effective when used alone or in combination with other chemotherapies in treating several malignancies, such as metastatic renal cell cancer,^10^ metastatic colorectal cancer,^11^ metastatic breast cancer,^12^ and metastatic nonsquamous nonsmall-cell lung cancer.^13^ In patients with advanced gastric cancer, several phase II studies suggest that the addition of bevacizumab improves the efficacy of chemotherapy, such as oxaliplatin, docetaxel, cisplatin, and fluorouracil.^14,15^ Although the initial results of the phase III study AVAGAST did not show beneficial effects of bevacizumab for patient survival as the first-line treatment for advanced gastric cancer,^16^ further analysis of the data suggest that certain subgroups may have benefited from the addition of bevacizumab to chemotherapy.

The docetaxel/oxaliplatin/5-FU (DOF) regimen has shown good efficacy in the treatment of advanced gastric cancer.^17,18^ In this study, patients with LAGC were randomized to receive DOF regimen (docetaxel/oxaliplatin/5-FU/calcium folinate) or DOF plus bevacizumab as neoadjuvant chemotherapy before surgical resection of the tumor. The patients were compared in terms of R0 resection rate and survival rate. We also examined the circulating tumor cells and analyzed its role in the mechanisms of neoadjuvant therapy.

## MATERIALS AND METHODS

### Patients

From December 2009 to June 2013, 80 patients with LAGC were included in this prospective, randomized, open-label study. The inclusion criteria were: pathological diagnosis of gastric adenocarcinoma; stages of T3–4, N1–3, M0; with possibly resectable lesion; Karnofsky score > 80. Patients with distal metastases were excluded from this study. This study was approved by the Ethics Committee of our hospital. Informed consent was obtained from all patients.

### Neoadjuvant Regimens

A total of 80 patients were randomly assigned into 2 groups using a random table and received DOF regimen (n = 40) or DOF plus bevacizumab (n = 40) as neoadjuvant chemotherapy before surgery. The DOF regimen included docetaxel 75 mg/m^2^ d 1 (intravenous drop), oxaliplatin 85 mg/m^2^ d 1 (intravenous drop for 2 h), calcium folinate 200 mg/m^2^ d 1–2 (intravenous drop), 5-FU 600 mg/m^2^ d 1–2 (infusion pump), and 5-FU 400 mg/m^2^ d 1–2 (bolus injection). In the DOF plus bevacizumab group, bevacizumab 5 mg per kg body weight was administered on day 1. Each cycle was 21 days. Four cycles were administered preoperatively and 2 cycles postoperatively. The lesions were evaluated at baseline and every 8 weeks during treatment according to RECIST 1.1 using computed tomography and endoscopic ultrasonography. If no response was seen on computed tomography images, the DOF regimen was replaced with the IFL regimen (irinotecan 125 mg/m^2^ replacing docetaxel and oxaliplatin, plus 5-FU 500 mg/^2^). In the DOF plus bevacizumab group, there was a 4-week interval between the neoadjuvant therapy and surgery.

### Treatment Evaluation

All measurable lesions were evaluated using the maximum diameter. Complete remission was defined as disappearance of the lesion lasting at least 4 weeks. Partial remission was defined as shrinkage of the lesion >30% lasting at least 4 weeks. Disease progression was increase of the lesion >20%. Other patients with lesions between partial remission and disease progression were defined as stable disease. Adverse events were recorded according to the Common Terminology Criteria for Adverse Events (CTCAE) 4.0 criteria.

All surgeries were performed by the same team. Pathologic complete remission was confirmed if the resected sample had no tumor or only had in situ lesions. All patients were followed up for 3 years. The primary endpoint was the R0 resection rate. The secondary endpoints were pathologic complete remission, disease-free survival (DFS), and overall survival (OS).

### Examination of Circulating Tumor Cells

The circulating tumor cells (CTCs) were counted before neoadjuvant therapy, after neoadjuvant therapy, before surgery, and after surgery. At each time point, 7.5 ml peripheral blood was collected from each patient into tubes containing EDTA. The red blood cells were lysed and CD45 + cells were removed using immunomagnetic beads. A total of 1 × 10^4^ cells were subjected for FISH for the centromeres of chromosomes 7 and 8 for the identification of CTCs. Those cells with >2 copies of chromosomes 8 or 17 were regarded CTCs.

### Statistical Analysis

Continuous data were presented as mean ± standard deviation. Categorical data were presented as frequencies or percentages. Comparisons were made using Student's *t* test for continuous data and the χ^2^ test for categorical data. Pearson's correlation analysis was performed to analyze the correlation between CTC counts and DFS. Statistical analyses were performed using SPSS 17.0 software (SPSS Inc, Chicago, IL). The Cox proportional hazards model was used to analyze the prognostic factors of patients with LAGC. A *P* value <0.05 was considered statistically significant.

## RESULTS

### Patient Information

The patient information at baseline is listed in Table 1. No significant differences were found in age, sex, tumor size, and disease stage between the 2 groups.

**TABLE 1:**
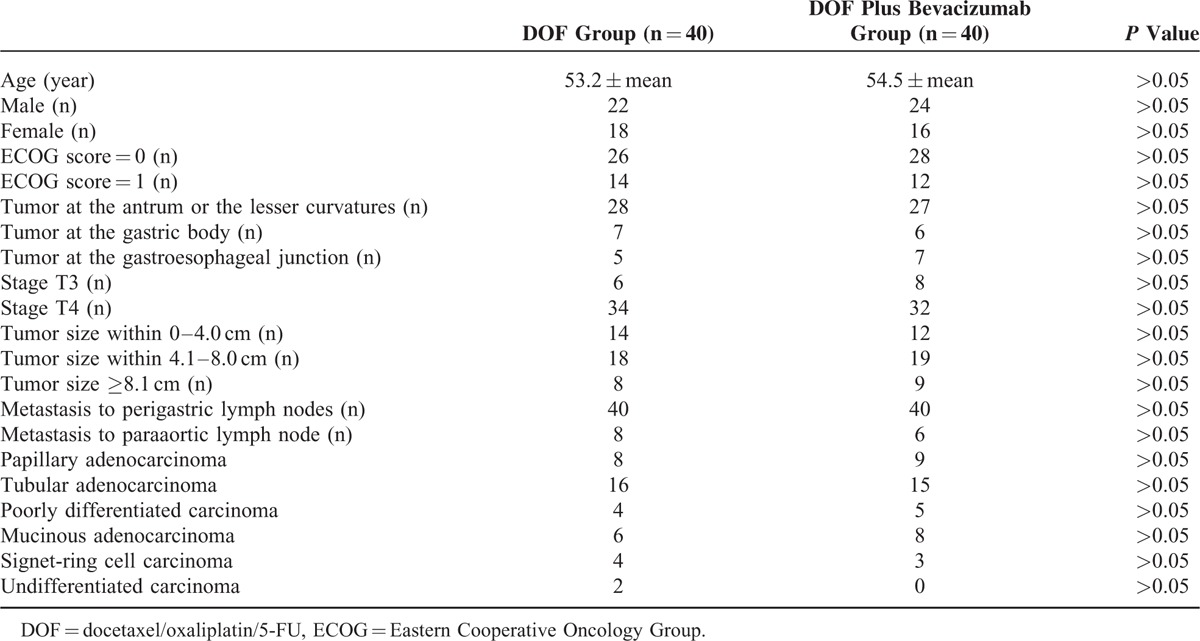
Baseline Information of the DOF Group and the DOF Plus Bevacizumab Group

### Efficacy of the Neoadjuvant Therapy

In the DOF group, there were 2 patients with complete remission and 15 patients with partial remission. In the DOF plus bevacizumab group, there were 4 patients with complete remission and 22 patients with partial remission (Table 2). The total response rate (complete and partial remission) was significantly higher in the DOF plus bevacizumab group than the DOF group (65% vs 42.5%, *P* = 0.0436).

**TABLE 2:**
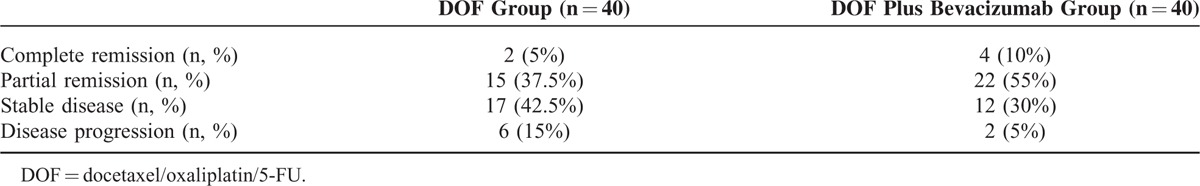
Treatment Efficacy of the Neoadjuvant Therapy in the DOF Group and the DOF Plus Bevacizumab Group

### Surgical Results

Significantly more patients received D2 surgery in the DOF plus bevacizumab group than the DOF group (77.5% vs 52.5%, *P* = 0.0191) (Table 3). The R0 resection rate was significantly higher in the DOF plus bevacizumab group than the DOF group (75% vs 50%, *P* = 0.0209). There were 2 patients with pathologic complete remission in the DOF group and 4 patients in the DOF plus bevacizumab group.

**TABLE 3:**
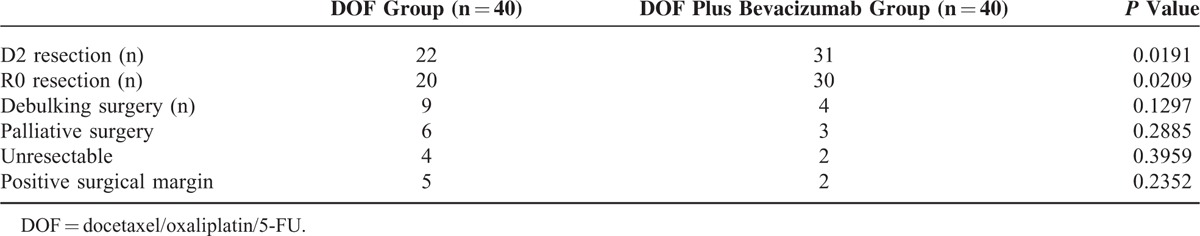
Surgical Results of the DOF Group and the DOF Plus Bevacizumab Group

### CTC Counts

**FIGURE 1:**
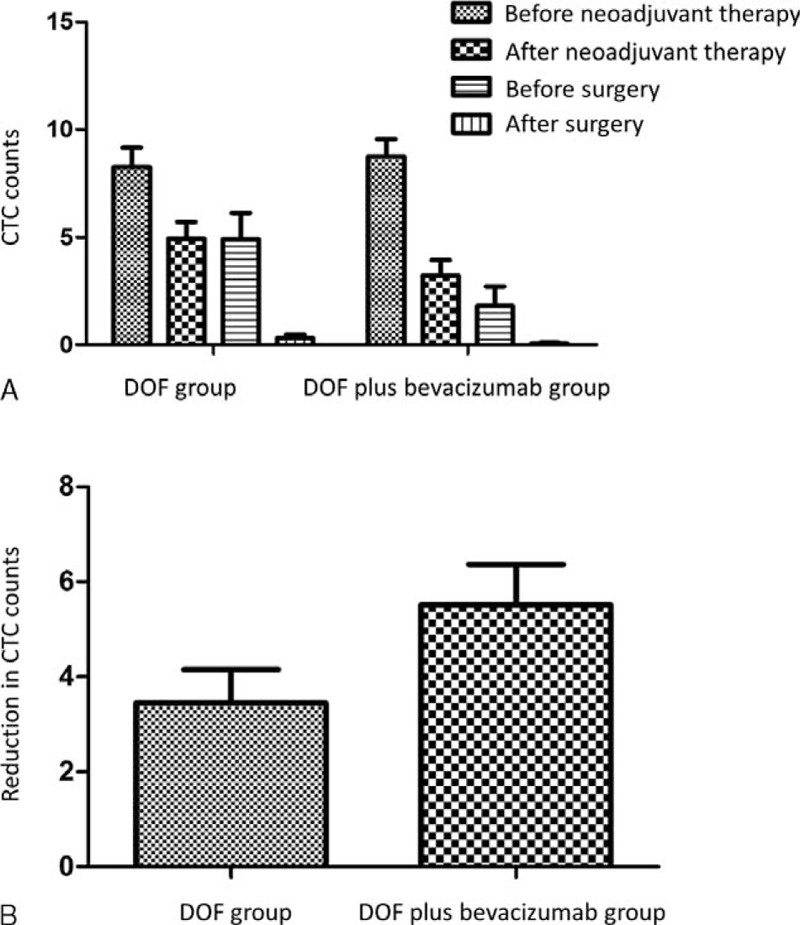
CTC counts in the DOF group and the DOF plus bevacizumab group. (A) Neoadjuvant therapy and surgery significantly decreased CTC counts in both groups. (B) The DOF plus bevacizumab group showed significantly higher reduction in CTC counts than the DOF group (*P* = 0.0335). CTC = circulating tumor cells, DOF = docetaxel/oxaliplatin/5-FU.

At baseline, 37 patients (92.5%) in the DOF group and 38 patients (95%) in the DOF plus bevacizumab group were positive of CTCs. In both groups, the CTC counts were significantly decreased after neoadjuvant therapy and surgery (Figure 1A). However, the reduction in CTC counts after neoadjuvant therapy was significantly greater in the DOF plus bevacizumab group than the DPF group (Figure 1B, *P* = 0.0335). This suggests that the addition of bevacizumab can further decrease the CTC counts.

### Survival Analysis

**FIGURE 2:**
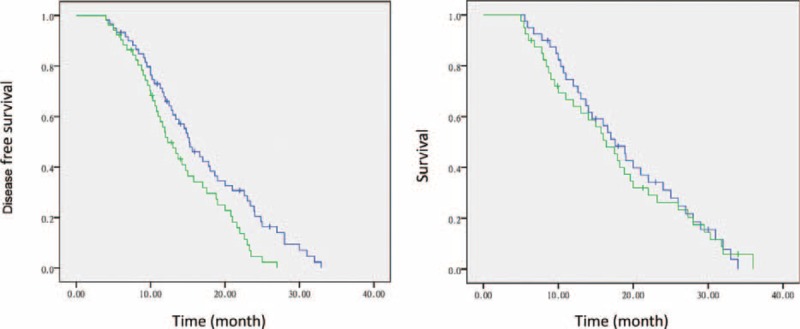
The DOF plus bevacizumab group had significantly longer DFS than the DOF group (*P* = 0.013). However, the median survival time did not differ significantly between the 2 groups (*P* = 0.776). Blue curve: DOF plus bevacizumab group. Green curve: DOF group. DOF = docetaxel/oxaliplatin/5-FU, DFS = disease-free survival.

The DOF plus bevacizumab group had significantly longer DFS than the DOF group (15.2 months vs 12.3 months, *P* = 0.013). During the 3-year follow-up, the median survival time did not differ significantly between the 2 groups (16.4 ± 1.9 years in the DOF group, 95% CI: 12.9–19.9; 17.6 ± 1.8, 95% CI: 14.2–21.0; *P* = 0.776) (Figure 2).

### Correlation Analysis

**FIGURE 3:**
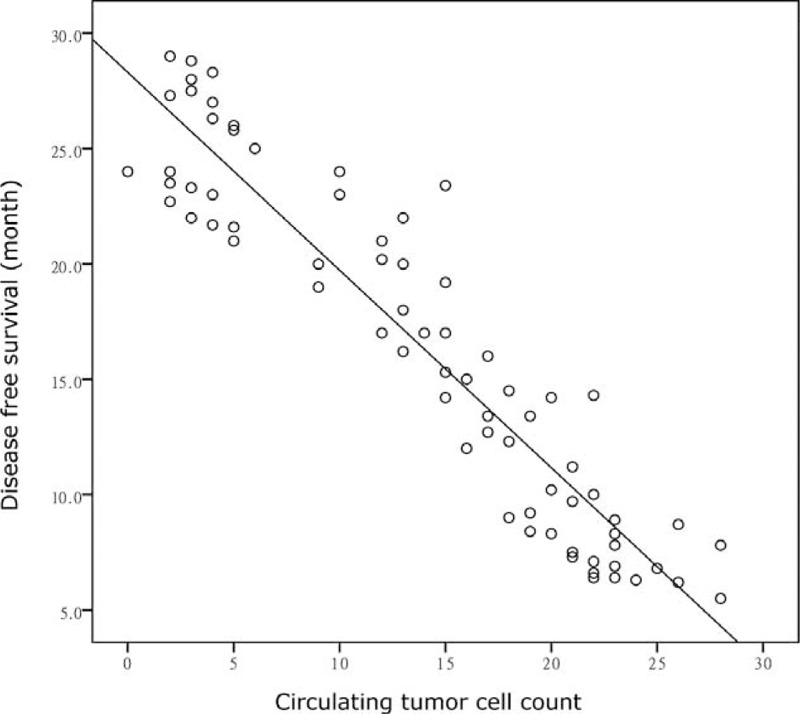
Pearson's correlation analysis showed that DFS was positively correlated with CTC counts (*r* = −0.93, *P* < 0.05). DFS = disease-free survival, CTC = circulating tumor cells.

In all 80 patients, Pearson's correlation analysis showed that DFS was positively correlated with CTC counts (*r* = −0.93, *P* < 0.05; Figure 3).

### Adverse Events

The most common adverse events were nausea, vomiting, sensory neuropathy, and leukopenia. Other adverse events were mostly grate 1–2, such as decreased hemoglobin, thrombocytopenia, mucositis, and diarrhea. No significant difference was noticed in frequencies of adverse events between patients receiving DOF or DOF plus bevacizumab.

### Prognostic Factors

Univariate and multivariate analyses found that age, sex, pathological type, and infiltration depth are not significantly associated with LAGC patient prognosis (all *P* > 0.05). However, number of metastatic lymph nodes (95% CI, 1.563–7.037), CTC reduction (95% CI, 1.208–5.076, R0 resection (95% CI, 1.133–3.512), and neoadjuvant therapy (95% CI, 0.224–0.707) were found to be independent prognostic factors.

## DISCUSSION

This clinical trial showed that the addition of bevacizumab to the DOF neoadjuvant chemotherapy for LAGC patients can significantly increase the D2 surgery rate and the R0 resection rate. Bevacizumab in combination with DOF also significantly increased DFS than DOF alone. However, the increased R0 resection rate did not translate into better patient OS. Furthermore, the addition of bevacizumab produced greater reduction in CTC counts than DOF alone, which might be associated with the higher R0 resection rate in the DOF plus bevacizumab group.

Most Chinese patients with gastric cancer present at advanced stages of III or IV. Only about half of these advanced gastric cancer is resectable. In addition, these patients often have subclinical metastases and tend to have recurrence after surgical resection of the tumor, and therefore have poor prognosis. In recent years, many new drugs or new combination of drugs have shown good efficacy and tolerance in treating advanced gastric cancer, and have been used in neoadjuvant chemotherapy for this disease.^19–28^ There are several proposed mechanisms for the neoadjuvant therapy. Preoperative chemotherapy can shrink the tumor, lower tumor burden, downgrade the clinical staging, and improve the surgical resection rate. Neoadjuvant chemotherapy also controls the microcarcinomas and subclinical lesions, and inhibits tumor cell proliferation stimulated by surgical manipulation, therefore decreasing the recurrence rate. Infusion of drugs before the disruption of tumor vasculature and lymphatic vessels can produce higher drug concentration and better efficacy. Preoperative chemotherapy also allows for assessing the sensitivity to postoperative chemotherapy. Finally, neoadjuvant therapy can decrease the risk of iatrogenic implantation and distal metastasis.

Chemotherapies containing 5-FU and its derivatives in combination with cisplatin-based drugs and taxanes or anthracycines are still the mainstay for gastric treatment. The DOF regimen used in this study was modified from the DCF regimen (docetaxel, cisplatin, and fluorouracil). The DCF regimen has shown good efficacy in postoperative chemotherapy for gastric cancer, but its use in neoadjuvant therapy is still scarce. Guo et al treated 24 patients with T4 stage gastric cancer using DCF as neoadjuvant therapy and achieved the R0 resection rate of 77% and 3-year survival rate of 79%.^29^ In a phase I/II trial with 59 patients with localized carcinoma of the esophagus or gastroesophageal junction, preoperative DOC regimen in combination with radiotherapy produced pathologic complete remission rate of 49% and objective response rate of 61%.^30^ Sym et al treated 49 gastric cancer patients using the DXP regimen as neoadjuvant therapy and achieved the R0 resection rate of 63% and DFS of 54 months in patients with R0 resection.^31^ In our study, the addition of bevacizumab to the DOF regimen as neoadjuvant therapy further improved the surgical resection rate compared with DOF alone. The combined use of bevacizumab and DOF regimen did not significantly increase the adverse events. In addition, 4 patients in the DOF plus bevacizumab group had pathologic complete remission, suggesting that this combination can effectively improve pathologic remission.

In conclusion, the addition of bevacizumab to the DOF neoadjuvant chemotherapy for LAGC patients can significantly increase the R0 resection rate and DFS. The beneficial effects of this neoadjuvant regimen might be associated with the reduction in CTC counts.
